# Theory and Practice of the Movement-Cure

**Published:** 1861-09

**Authors:** 


ART. IX.—*Theory and Practice of the Movement-Cure; or, the Treatment of Lateral Curvature of the Spine, Paralysis, Indigestion, Constipation, Consumption, etc., by the Swedish System of Localized Movements.* By CHARLES FAYETTE TAYLOR, M.D. With Illustrations. 12mo., pp. 295. Philadelphia: Lindsay & Blakiston, 1861.

WE take up this work for notice with some distrust of our being able to forget, for the time being, that it looks like an advertisement of a specialty. It is an attempt to introduce a new mode of practice, and to apply it to a large number of the diseases included in the catalogue of human ills. Like all such innovations on rational therapeutics, its merits, whatever they are, may be pushed further than probability of success would justify. A practitioner who sees good effects in isolated cases is often disposed to generalize and apply his modes of treatment universally. This book of gymnastic exercises appears as if it might be placed under the same category. The Movement-Cure, under its various synonyms, Kinesipathy, Kinesitherapia, Motorpathy, Medicina Mechanica, and Lingism, is not of very recent introduction into medical arts, having been applied, in the words of Dr. Taylor, to the cure of diseases "by Peter Henry Ling, poet and philosopher, to teach us not only to despise effeminacy, and to emulate the physical nobleness of the old Norse heroes, but to banish disease by the beautiful system he originated."

We do not stop here to discuss the theoretical views of the author, or to refer to the intimate connection, which he dwells upon, between muscular action and the exercise of various functions in health and disease. The therapeutical portion of this collection of monographs, which were originally published in the medical journals of New York, constitutes the special subject of our consideration. The principles of treatment may be best explained in the language of the writer:—

"The Movement-Cure treatment," he says, "is simply certain physiological principles put to practice. That, by a right employment of the muscles, we can produce *mechanical* results and alter the form of the body, can be illustrated in the treatment of spinal curvatures and other deformities; that we can produce, by a modification of the same means, a special impression on the nervous system, can be seen in the treatment of paralysis; and that the general physiological processes can be wholesomely affected, will be shown in the treatment of indigestion, consumption, and various other chronic affections."

The author attempts to show, in the early chapters, how, by proper localized movements, in contradistinction to irregular efforts, which might hinder or interfere with, instead of assisting, a depressed function or organ, certain special physiological purposes may be accomplished as a direct result; and how designated groups of muscles, as in lateral curvature of the spine, may be developed, independently of other muscles, so as to cause a muscular action with reference to the spinal column, opposite that which produced the curvature, with the effect of ameliorating or curing the deformity.

> "Even the nerve of a paralyzed muscle," he says, "may be aroused by assuming the conditions necessary for volition to reach it, and it is possible to re-educate it to something like its former functional capacity. So much, then, being under our intelligent control, when managed so as to correct abnormal conditions, becomes, in fact, a medical treatment in those particular cases."

With enthusiastic views of success, the author confidently states that "we may secure the most extensive voluntary sway over the whole system, and its several parts, through the influence which regulated muscular action has over the circulation of the blood," and applies this system of therapeutics to the treatment of constipation, chronic diarrhœa, dyspepsia, pulmonary consumption, angular curvature of the spine, or "Pott's Disease," deformities of the limbs, diseases incident to women, and derangements of the nervous system. One great objection, we should think, to the employment of kinesipathy, in some of these debilitated cases, would be that the remedy, so powerful in its action, might be worse for the patient than the disease; but Dr. Taylor says not, and cites cases to support his enthusiastic views.

